# Alternative techniques for cardiopulmonary resuscitation in extreme environments − A scoping review

**DOI:** 10.1016/j.resplu.2024.100762

**Published:** 2024-09-03

**Authors:** Remco Overbeek, Felix Liebold, Lydia Johnson Kolaparambil Varghese, Niels-Benjamin Adams, Jan Schmitz, Michael Neumann, Fabian Dusse, Sandra E. Stoll, Wolfgang A. Wetsch, Jochen Hinkelbein

**Affiliations:** a Department of Anesthesiology and Intensive Care Medicine, Faculty of Medicine and University Hospital Cologne, University of Cologne, Cologne, Germany; b German Society of Aerospace Medicine (DGLRM), 80331 Munich, Germany; c Department of Anesthesiology and Intensive Care Medicine, University Hospital of Leipzig, 04103 Leipzig, Germany; d Department of Anaesthesiology, Intensive Care, Pain Medicine and Emergency Medicine, Johannes-Wesling-Universitätsklinikum Minden, Ruhr-Universität Bochum, Minden, Germany; e Department of Anesthesiology, Montefiore Hospital, Albert Einstein University, NY, USA

**Keywords:** Advanced life support, Chest compression, CPR, Out-of-hospital cardiac arrest

## Abstract

**Background:**

Cardiopulmonary resuscitation (CPR) is essential for saving lives during cardiac arrest, but performing CPR in extreme environments poses unique challenges. In scenarios ranging from hypogravity or microgravity to confined spaces like aeroplanes and underwater scenarios, traditional CPR techniques may be inadequate. This scoping review aims to identify alternative chest compression techniques, synthesise current knowledge, and pinpoint research gaps in resuscitation for cardiac arrest in extreme conditions.

**Methods:**

PubMed and the Cochrane Register of Controlled Trials as well as the website of ResearchGate was searched to identify relevant literature. Studies were eligible for inclusion if they evaluated alternative chest compression techniques, including manual or mixed CPR approaches, whilst assessing feasibility and effectiveness based on compression depth, rate, and/or impact on rescuer effort.

**Results:**

The database search yielded 9499 references. After screening 26 studies covering 6 different extreme environments were included (hypogravity: 2; microgravity: 9, helicopter: 1, aeroplane: 1, confined space: 11; avalanche: 2). 13 alternative chest compression techniques were identified, all of which tested using manikins to simulate cardiac arrest scenarios.

**Conclusion:**

To address the unique challenges in extreme environments, novel CPR techniques are emerging. However, evidence supporting their effectiveness remains limited.

## Introduction

The delivery of high-quality cardiopulmonary resuscitation (CPR) is critical for survival of individuals experiencing cardiac arrest.^1^ Current guidelines emphasize the importance of appropriate depth (50–60 mm) and rate (100–120 min^−1^) of external chest compressions. This is crucial to ensure an adequate cardiac output to perfuse vital organs and achieve return of spontaneous circulation (ROSC).^2^

While well-established standard techniques for chest compressions prevail in traditional medical settings, the necessity for innovative approaches becomes apparent when confronted with environments that deviate from the ordinary conditions found in hospitals. Cardiac arrest in these unconventional settings presents distinctive challenges, necessitating a deviation from conventional resuscitation methods to achieve adequate CPR quality.^3^

Extreme environments include environments defined by microgravity or hypogravity, experienced in space exploration scenarios, which introduce significant obstacles to the effective delivery of chest compressions due to the absence or lack of gravity.^4^, ^5^ Similarly, CPR in confined spaces like aeroplanes or helicopters add complexities that demand adaptations to traditional CPR protocols.^6^ Avalanche situations, marked by the burial of individuals under layers of snow, require considerations for both the challenging physical environment of high altitude, limited patient access and the potential trauma sustained during the event.^7^ In aquatic settings, where drowning incidents are prevalent, the efficacy of chest compressions is influenced by the buoyancy of the body and the intricacies of performing CPR in water.^8^ Recognizing the critical importance of timely and effective chest compressions as part of CPR in these extreme scenarios, researchers have explored alternative techniques to address the unique challenges posed by each environment. The development and evaluation of innovative chest compression techniques, devices, protocols, and training methodologies have gained prominence in recent years.^2^, ^3^, ^5^, ^9^ These alternative techniques aim not only to optimize the chances of successful resuscitation but also to minimize the potential complications associated with performing CPR in conditions that deviate from the norm.

This scoping review aims to identify alternative chest compression techniques, synthesizes the current state of knowledge, and identifies research gaps in resuscitation tailored to the challenges posed by cardiac arrest in extreme conditions.

## Method

We have conducted a comprehensive literature search using a predefined search algorithm developed by RO, FL and JH. Keywords were selected after literature review and based on the authors expertise in the field to cover all areas of out-of hospital emergencies, where traditional CPR methods are impractical. Two independent reviewers (RO and FL) systematically searched MEDLINE via PubMed, the Cochrane Register of Controlled Trials as well as the website of ResearchGate to identify relevant studies. All retrieved studies were organized and managed using the EndNote reference management software (EndNote 20 for Macintosh, Clarivate Analytics, Philadelphia, USA). In the final screening process reviewers were assigned different topics for full-text screening. Regular meetings by video conference were held to monitor progress and discuss potential eligible studies. When in doubt a final decision was made by RO and FL. The reference lists of included studies were also searched to identify relevant articles that may have been missed. The screening process and report of this review is in accordance with the Preferred Reporting Items for Systematic reviews and Meta-Analyses extension for Scoping Reviews.^10^ A review protocol for this study was not published beforehand.

Our inclusion criteria were: studies focusing on out-of-hospital cardiac arrest scenarios examining chest compression techniques with or without ventilation. We considered studies involving manual or mixed manual/mechanical CPR with non-traditional techniques, positions, or algorithms. Studies had to present data on the feasibility and/or effectiveness of these techniques, such as information regarding compression depth, rate, or the impact on rescuer effort and fatigue. Our criteria encompassed both controlled and non-controlled trials. Conversely, studies were excluded if they were not available in English, lacked full-text versions, or did not present relevant data. Additionally, studies involving children were excluded from our analysis. Narrative reviews and animal studies were also excluded. No studies were excluded from the screening based on their publication date. Grey literature was not screened.

Details pertaining to the study environment, research methodology, demographic attributes of the population, types of interventions, comparisons made, outcomes measured, and significant findings were systematically documented on a standardized data collection form. No formal critical appraisal of study quality was undertaken.

Search algorithm:

(CPR OR resuscitation OR ALS OR BLS OR chest compression) AND (microgravity OR hypergravity OR space OR zero G OR hypogravity OR boat OR confined space OR aeroplane OR helicopter OR hyperbaric OR diving bell OR avalanche OR extreme environment OR open water OR straddling position OR over the head OR adapted technique).

## Results

**Fig. 1:**
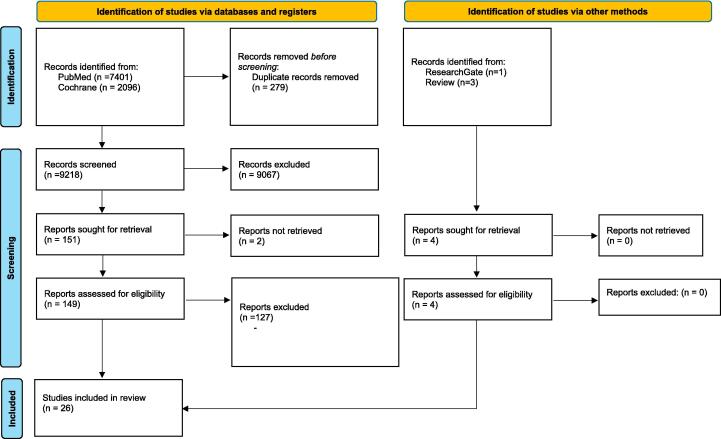
Prisma Flow Chart.

The database search was conducted in June 2023 and yielded 9,497 references. After removal of duplicates, 9,218 papers were left for screening titles and abstracts. Three studies were identified through other reviews and one through the website ResearchGate. The number of papers identified for full text review and final selections are summarised in Fig. 1. Finally, 26 references were found eligible to be included in this review. All relevant information on study design, participants, setting, alternative technique and outcomes is summarised in Table 1. In total 13 alternative chest compression techniques were identified, all of which tested using manikins to simulate cardiac arrest scenarios.

**Table 1:** Summary of the characteristic of the studies included in the scoping review on the resuscitation in extreme environments, EMT = Emergency medical technician, ERC: European Resuscitation Council, AHA: American heart association, CPR: Cardiopulmonary resuscitation, OTH: Over the head, ILCOR: International Liaison Committee on Resuscitation, MRD: Mechanical resuscitation device, **°** Effort measured by heart rate or BORG scale.

**Country**	**Publication**	**Year**	**Participants**	**Design**	**Setting**	**Technique**	**Protocol**	**Control**	**Outcome**		
**1.Hypogravity**									**Depth**	**Rate**	**Effort°**
Brazil	Mackaill et al.^9^	2018	10 participants	Repeated Measures Design	Body-Suspension Device simulating 0,17G and 0,38G	Mackaill–Russomano	3 × 30 compressions only	Traditional Technique (ERC 2015)	X.	X	X
France	Benyoucef et al.^11^	2014	One investigator	Repeated Measures Design	Modified cable-cross over fitness machine simulating 0,16G and 0,39G	Seated-Armlock	3 min (30 compressions + 6 s break)	Traditional Technique (AHA 2010)	X	X	X
**2. Microgravity**											
USA	Jay et al.^12^	2003	8 participants	Observational Comparative Study	Parabolic flight on the KC-135A	Handstand, standard side straddle, waist straddle maneuver, Reverse Bear Hug	5:1 compressions/ventilation with two rescuers	+1 G Traditional Technique (AHA 2000)	X	X	
Great Britain	Rehnberg et al.^13^	2011	21 male subjects	Repeated Measures Design	Full body suspension	Evetts-Russomano	3 min (30 compressions + 6 s break)	+1 G Traditional Technique (AHA 2005)	X	X	X
Germany	Schmitz et al.^14^	2022	15 paramedics	Randomized, controlled trial	Submerged underwater setting	Schmitz-Hinkelbein- Method and Cologne-Method	1 min CPR	+1 G Traditional Technique (ERC 2021)	X	X	
Great Britain	Evetts et al.^15^	2005	Two investigators	Repeated Measures Design	Parabolic flight	Evetts-Russomano	22 s compressions compared with compression plus ventilation	+1 G Traditional Technique (ERC 2000)	X	X	
Great Britain	Rehnberg et al.^16^	2014	23 male subjects	Randomized Repeated Measures Design	Body suspension device	Evetts-Russomano, Handstand and Reverse Bear Hug	4 x 1.5 min (30 compressions + 6 s break)	+1Gz Traditional Technique (ERC 2010)	X	X	X
Great Britain	Kordi et. Al^17^	2011	10 male subjects	Randomized Repeated Measures Design	Body suspension device	Evetts-Russomano, Handstand and Reverse Bear Hug	3 x 1.5 min (30 compressions + 6 s break)	No control	X	X	X
Brazil	Russomano^18^	2013	30 male volunteers	Randomized Repeated Measures Design	Body suspension device	Evetts-Russomano	4 x 30 compressions	+1 G Traditional Technique (ERC 2005 and ERC 2010)	X	X	X
Brazil	Baptista^19^	2015	30 male volunteers	Randomized Repeated Measures Design	Body suspension device	Evetts-Russomano	3x 30 chest compressions	+ 1 G Traditional Technique (AHA 2010)	X	X	X
Great Britain	Kordi^20^	2012	20 mal and 12 female volunteers	Randomized Repeated Measures Design	Body suspension device	Evetts-Russomano	3x 30 chest compressions with 6 s rest	+ 1 G Traditional Technique (AHA 2010)	X	X	X
**3. Aeroplane**											
Austria	Fischer et al.^21^	2011	80 flight attendants	Prospective, open, randomized and crossover simulation study	Airbus A-320 cabin simulator, galley	MRD	2005 ERC, 30:2	Traditional Technique (2005 ERC)	X	X	
**4. Confined Space**											
Germany	Bollig et al.^22^	2007	8 paramedic students	Randomised double-crossover study	Manikin study	OTH	ERC 2000, 2 rescuers and two positions per CPR technique, four experiments per scenario = a total of 16 experiments	Traditional Technique (ERC 2000)	X	X	
Germany	Brucke et al.^23^	2007	20 2-rescuer teams (trauma anaesthesiologist + Paramedic)	Feasibility study	Manikin study	OTH	Especially developed ‘‘two rescuer ALS algorithm’’ with OTH CPR according to ERC 2005, 10 min per team	No	X	X	
Taiwan	Chi et al.^24^	2009	21 health care providers	Observational Comparative Study	Manikin Study	OTH	AHA 2005, 2x2 minutes with 15 min resting period	Traditional Technique (AHA 2005)	X	X	
Poland	Ćwiertnia et al.^25^	2019	38 paramedics	Observational Comparative Study	Manikin study	OTH	ERC 2015, 2x30 chest compressions	ERC and AHA Guidelines reference values	X	X	
UK	Handley et al.^26^	2004	19 airline employees	Simulation comparative study	Manikin study	Straddle CPR + OTH	15:2, 4x1 minute	Traditional Technique (ERC 2000)	X	X	
Germany	Hüpfl et al.^27^	2005	67 emergency medical technician students	Simulation cross-over study	Manikin study	OTH	15:2, 2x2 minutes	Traditional Technique (AHA 2000)	X	X	
Germany	Maisch et al.^28^	2011	102 paramedics and EMTs	Simulation cross-over study	Manikin study	OTH vs. standard vs. alternating CPR	ERC 2005, 5x2 minutes	Single OTH vs. single standard versus single alternating	X	X	
Germany	Maisch et al.^29^	2010	106 medical students	Simulation cross-over study	Manikin study	OTH	ERC 2005, 30:2, 3x2 minutes (1x OTH, 2xstandard)	Traditional Technique (ERC 2005) with two persons	X	X	
UK	Perkins et al.^30^	2004	20 BLS instructors	Cross-sectional comparative study	Manikin study	OTH	ERC 2000, 2x3 minutes	Traditional Technique (AHA 2000)	X	X	X
Thailand	Supatanakij et al.^31^	2021	124 emergency physicians, general practitioners, nurses, ambulance staff, and medical and paramedic students	Cross-sectional comparative study	Manikin study	Straddle CPR	AHA 2015, compressions only, max. 4 min	Traditional Technique (AHA 2015) with one person	X	X	X
Indonesia	Wirmando et al.^32^	2023	100 nurses	Comparative study	Manikin study	OTH	Single rescuer OTH 30:2 for 2 min	Traditional Technique (AHA 2015) with one person	X	X	
**5. Helicopter**											
Canada	Pompa et al.^33^	2020	18 clinicians	Randomized Crossover trial	Helicopter BK1117	Koch method	Two minutes continuous chest compressions	Traditional CPR Technique (AHA 2015)	X	X	X
**6. Avalanches**											
Canada	Wallner et al.^34^	2021	25 medical students	Prospective, randomised cross-over study	Manikin with restricted access simulated by wooden panels	OTH and straddle position	Five rescue breaths followed by five cycles of CPR 30:2	Traditional CPR Technique (ERC 2021)	X	X	
Canada	Abrams et al.	2020	30 Skipatroller	Non-randomized, crossover study	Manikin on a moving toboggan	Straddle position on moving toboggan	5 x 30 compressions only	Straddle position on stationary toboggan (ILCOR 2015)	X	X	

## Summary of evidence

### Hypogravity

**Fig. 2:**
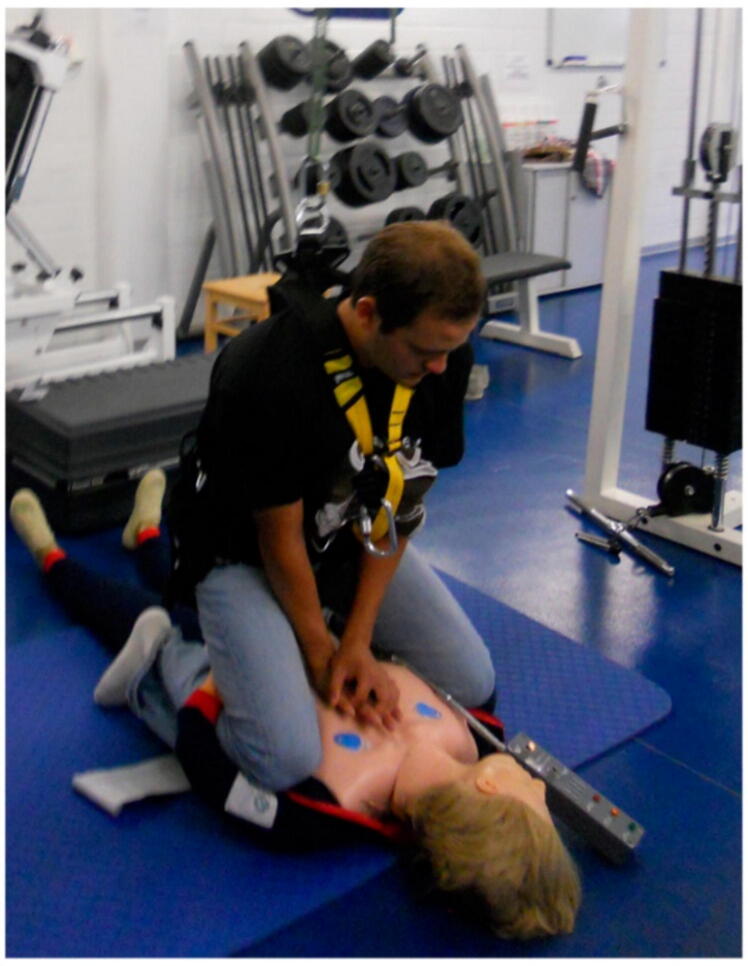
The Seated-Arm-Lock method.^4^

In our investigation, we identified two similar techniques: The Mackaill-Russomano method^9^ and the Seated-Arm-Lock-Method^11^(Fig. 2). In both approaches, the rescuer achieves stability either by locking the victim's arms behind their knees (Seated-Arm-Lock) or by tucking their heels and lower legs underneath the subject's legs (Mackaill-Russomano). These techniques were tested using body suspension devices to simulate lunar (0.16/0.17G) and Martian (0.38/0.39G) gravitational conditions. Results were then compared to the traditional technique recommended in current guidelines.

Mackaill et al.^9^ showed that the Mackaill-Russomano method demonstrated superior compression depths (47.4 ± 3.5 mm in 0.17G/ 50.5 ± 3.2 mm in 0,38G) compared to classic CPR (44.0 ± 3.5 mm in 0.17G/49.4 ± 3.4 mm in 0.38G) while showing similar or even reduced physical exertion of the rescuer measured by the rescuer’s heart rate. Benyoucef et al. demonstrated in a feasibility study with one participant that the Seated-Arm-Lock-Method achieved compression depths of 45 ± 6 mm (0.39G) and 42 ± 4 mm (0.16G), which were greater than the average compression depth of below 40 mm achieved with the traditional technique. Both authors emphasize the need for further studies confirming the results of these preliminary investigations.

### Microgravity

We have identified seven methods to perform CPR in microgravity which have been proposed in different approaches to simulate microgravity.

**Fig. 3:**
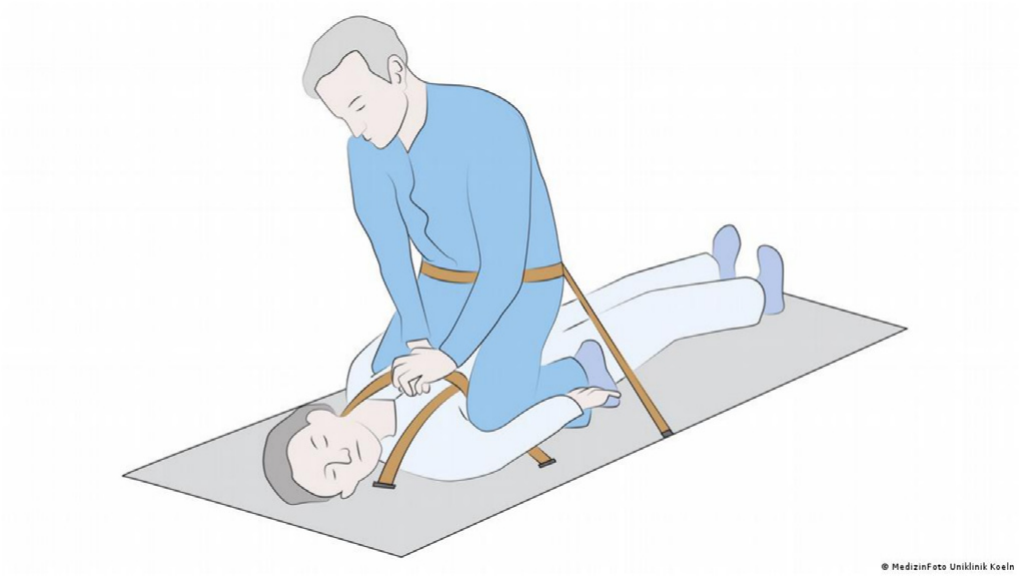
The Waist Straddling Maneuver (Image: MedizinFoto Uniklinik Koeln).
• The Standard Side Straddle technique involves the rescuer positioning themselves sideways, placing the patient on the crew medical restraint system for CPR^12^ (Fig. 4)

• In the Waist Straddling Maneuver, the rescuer straddles the patient's waist, utilizing the crew medical restraint system^12^ (Fig. 3)

**Fig. 4:**
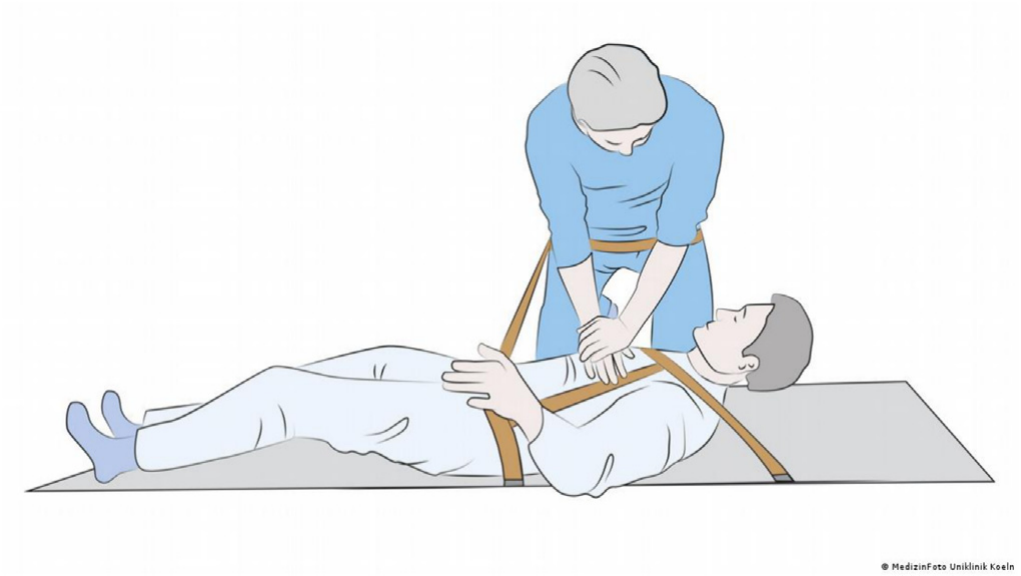
The Standard Side Straddle technique (Image: MedizinFoto Uniklinik Koeln).
• The Evetts-Russomano (ER) technique requires the practitioner to position themselves on top of the patient, crossing their legs over the patient's shoulder and back. The force exerted by the practitioner's crossed legs counters the chest compressions applied against the sternum^13^ (Fig. 5)

**Fig. 5:**
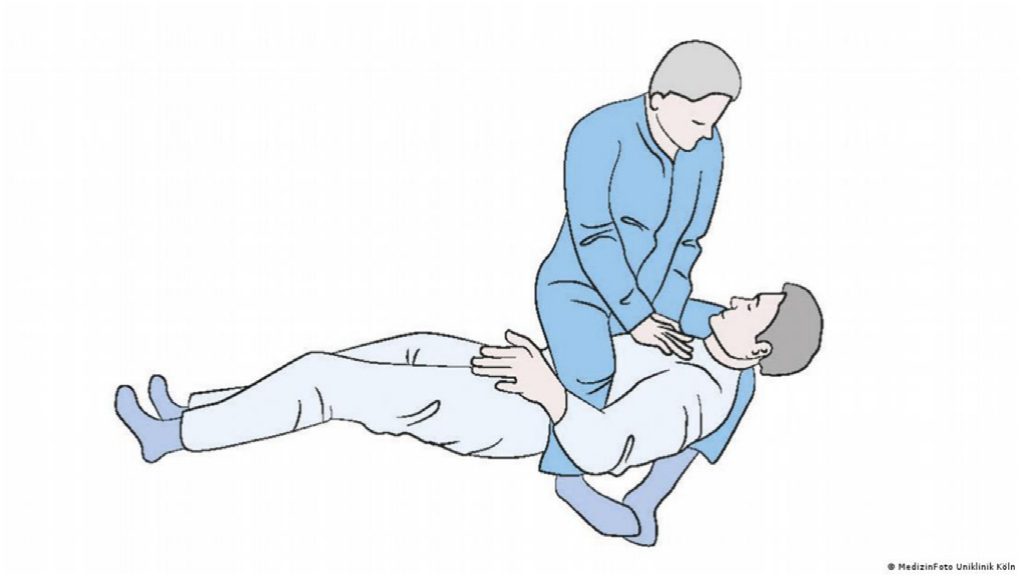
The Evetts-Russomano technique (Image: MedizinFoto Uniklinik Koeln).
• In the Reverse Bear Hug (RBH) approach, the rescuer embraces the patient from behind, using both arms to administer compressions^12^ (Fig. 6)

**Fig. 6:**
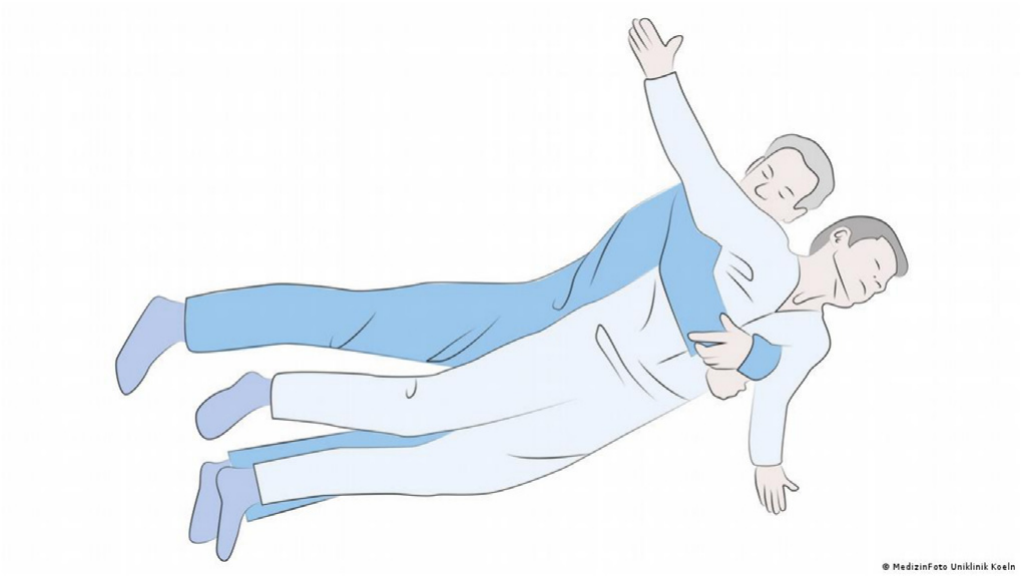
The Reverse Bear Hug technique (Image: MedizinFoto Uniklinik Koeln).
• For the Handstand method, the practitioner places their feet on one cabin wall while the patient's back is against the opposite wall. Therefore, it is recommended that spaceships be built with a wall-to-wall distance of no more than about two metres (Fig. 7)

**Fig. 7:**
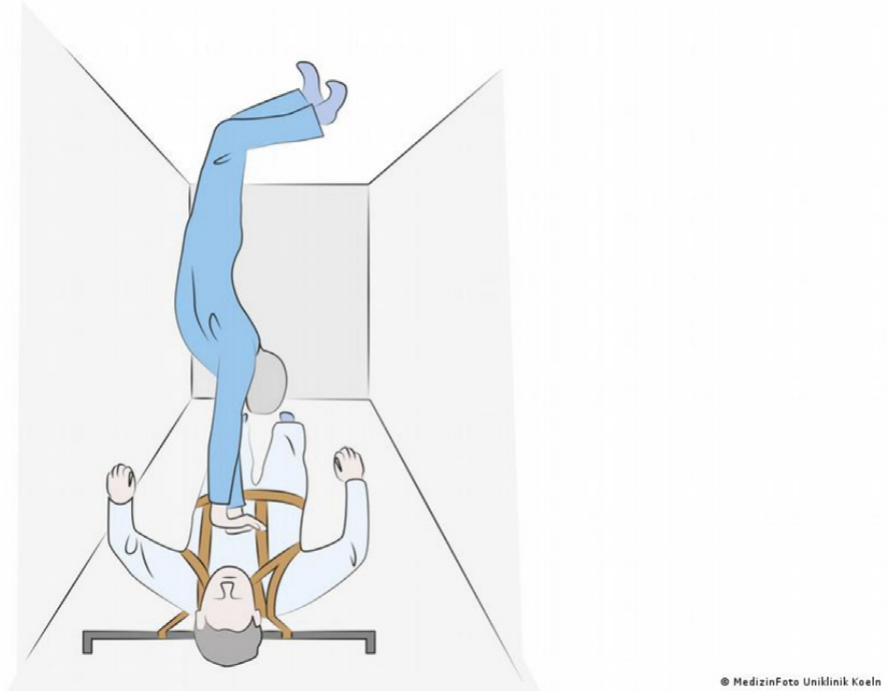
The Handstand technique (Image: MedizinFoto Uniklinik Koeln).
• Two techniques require the operator to stabilize the patient on his/her thighs and deliver chest compressions using both arms in the Schmitz–Hinkelbein method or using one elbow in the Cologne method^14^ (Fig. 8)

**Fig. 8:**
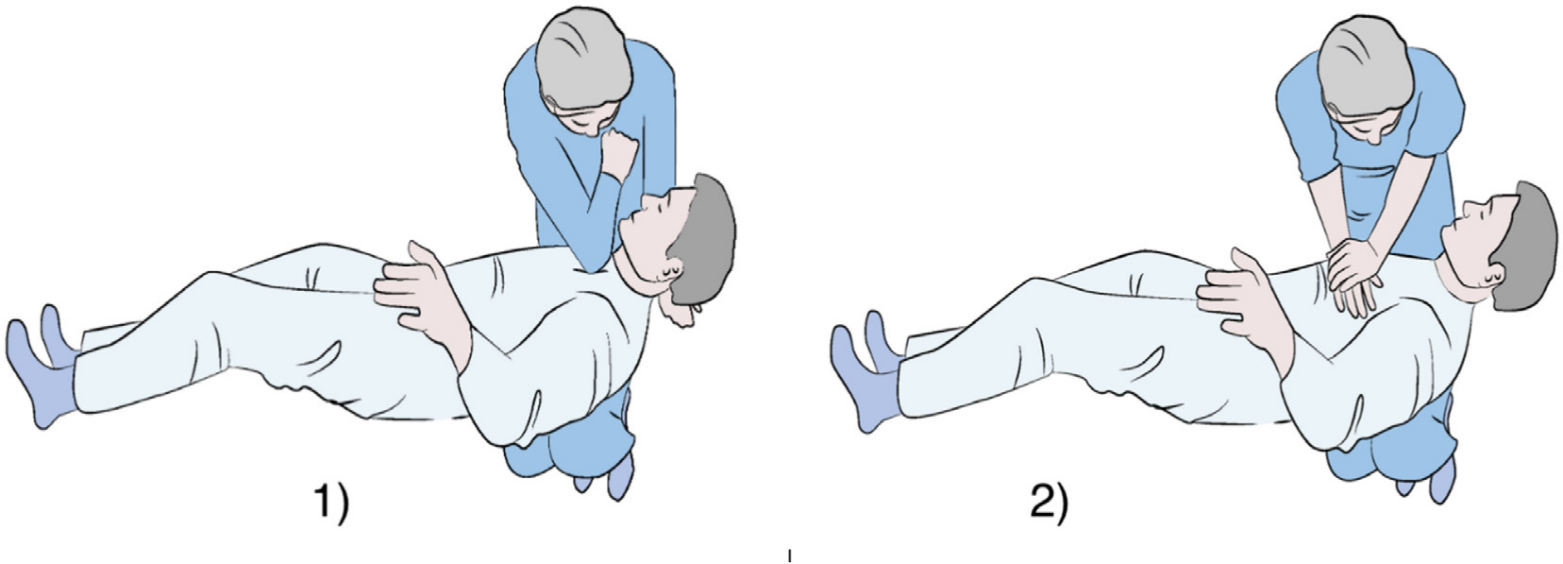
The Cologne method 1) and the Schmitz-Hinkelbein-method 2).^14^

The Waist Straddling Maneuver and Standard Side Straddle technique were tested by Jay et al. showing low levels of compression depth for the Standard Side Straddle technique (19.8 ± 11.2 mm) as well as the Waist Straddling Maneuver (30.7 ± 11.9 mm), while compression rates remained adequate. One study evaluating the ER method showed that effective chest compressions were not achievable in microgravity, especially regarding compression depth, which varied between 28.5 ± 7.0 mm and 32.9 ± 8.7 mm.^15^ Another study simulating microgravity with a full body suspension device reported, that the ER method can provide adequate compression depth (45.7 ± 2.7 mm) and rate (104.5 ± 5.2 compressions per minute) for CPR intervals up to three minutes.^13^ Due to the heightened physical demand of CPR in microgravity,^19^ female rescuers tend to fall below the recommended compression depth using the ER method.^20^

By using a full body suspension device Rehnberg et al. and Kordi et al. showed that the ER- and RBH-technique provided adequate compression rates but only the Handstand method met criteria for compression depth.^16^, ^17^ Jay et al. showed that during parabolic flight the Handstand method almost met current guidelines recommendation for compression frequency (98.3 ± 6.3 compressions per minute) and compression depth (40.01 ± 0.51 mm).^12^

The Schmitz-Hinkelbein method was tested in an underwater setting, showing superiority in compression rates (100.5 ± 14.4 compressions/min) with 65 ± 23 % of correct compression depths (and overall high rates of correct thoracic release after compression).^14^

Finally, a recent study reported, that mechanical chest compressions using an automated chest compression device (ACCD) allowed continuous delivery of high-quality CPR in microgravity, especially for compression depth (49.9 ± 0.7 mm).^36^

### Aeroplanes

**Fig. 9:**
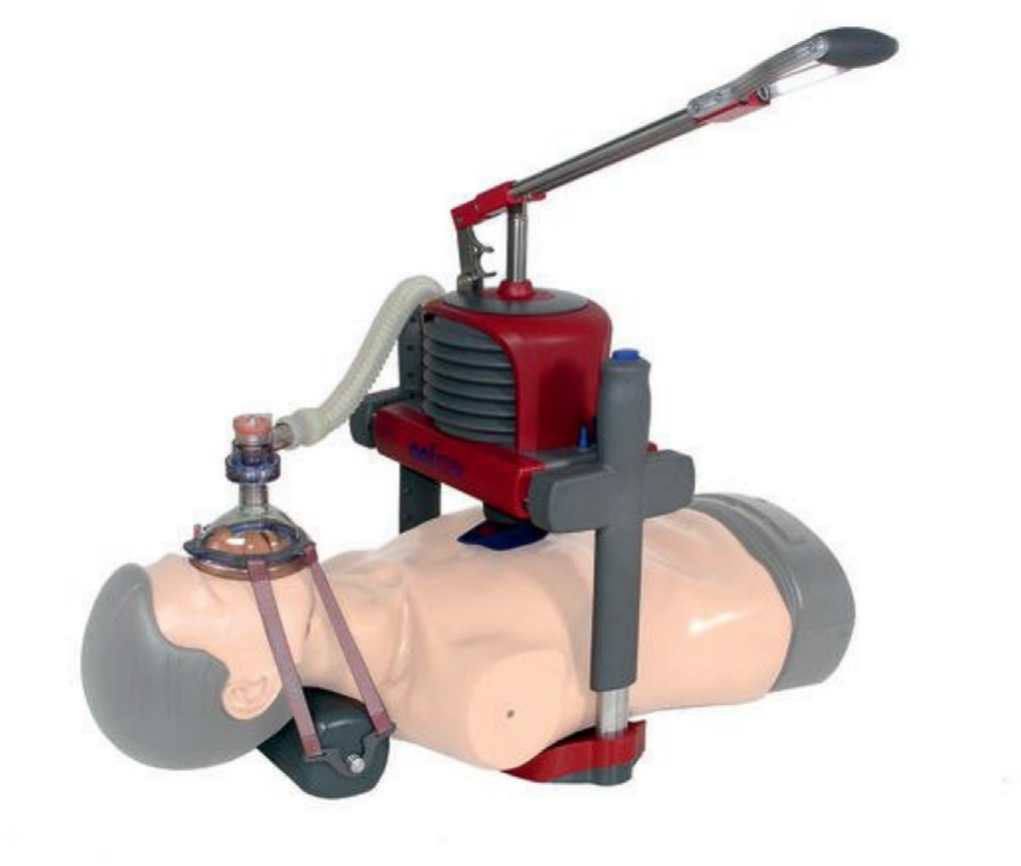
Animax (AAT Alber Antriebstechnik GmbH, Albstadt, Germany).

We identified one publication by Fischer et al. investigating an alternative CPR method in an aeroplane environment.^21^ The group performed a prospective simulation study to compare the quality of CPR applied by flight attendants between standard BLS and a new mechanical resuscitation device (MRD) called Animax (AAT Alber Antriebstechnik GmbH, Albstadt,

Germany) with an integrated mechanical intermediate ventilation function (Fig. 9). In conclusion, the authors stated that MRD resulted in significantly less absolute hands-off time (98 ± 47 s vs. 72 s ± 37 s) but at the same time less effective ventilation (107 ± 30 s vs. 92 ± 37 s).

### Helicopter

**Fig. 10:**
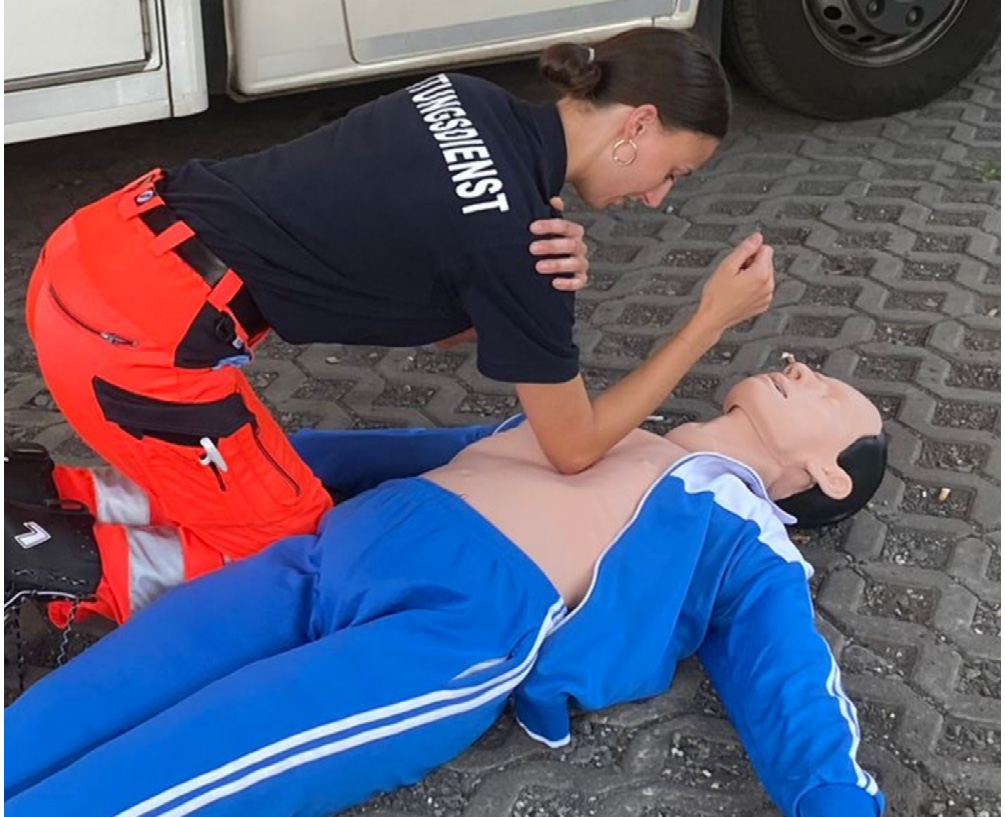
Simulation of the the Koch method on the ground; in a helicopter setting the second hand can be placed atop the forearm or used to brace oneself on the stretcher.^33^

We identified one publication investigating an alternative chest compression technique, the Koch method, developed specifically for performing CPR in helicopters.^33^ The study was conducted on a twin-engine medium utility helicopter, specifically the BK117 model. In the Koch method, the rescuer flexes one arm, positioning the proximal end of the radius and elbow on the inferior third of the patient's sternum. This technique, with the rescuers’ shoulder positioned above the elbow, facilitates effective transfer of upper body weight during compressions, optimizing CPR delivery (Fig. 10).

Comparative analyses demonstrated that Koch compressions exhibited higher overall quality scores (79 %) in contrast to conventional compressions (63 %). Koch compressions exhibited a mean depth of 51 mm, surpassing the conventional method by 5 mm on average. Evaluation of compression rates seem to reveal comparable performance between conventional and Koch compressions, with a mean rate of 112 beats per minute (bpm).

### Avalanches/High altitude

In this scoping review, two studies were found addressing the two major challenges of CPR in avalanche scenarios: The limited patient access^34^ and the ground-based transport during CPR.^35^

Abrams et al.^35^ aimed to compare the quality of CPR performed in a moving ski rescue sled with that of CPR performed in a stationary setting. In the stationary setting, a rescuer performed continuous chest compressions on a resuscitation manikin. Conversely, in the mobile resuscitation setting, a skier and a rescuer formed a functional unit. Here, the resuscitation manikin, secured with straps, lay on the ski sled with its head oriented towards the end, while the rescuer, adopting a kneeling position atop the manikin, performed chest compressions as the skier navigated the sled downhill.

In stationary conditions, the average compression depth reached 5.64 cm, with 97 % of compressions falling within the optimal depth range of 5–6 cm. Conversely, during mobile resuscitation, the mean compression depth decreased to 50.2 mm. Additionally, 86 % of chest compressions achieved complete thoracic decompression in stationary settings, whereas in mobile scenarios, 81 % were deemed complete. Remarkably, neither setting witnessed interruptions in chest compressions exceeding the stipulated 10-second threshold, underscoring the efficacy of CPR delivery in both stationary and mobile contexts.

In a randomized prospective crossover study, Wallner et al. investigated the one-man resuscitation technique in the context of avalanche burials.^34^ The study compared the standard CPR position with overhead resuscitation (OTH) and chest compressions in a straddled position above the patient, with mouth-to-mouth or pocket mask ventilation.

Across all positions, 98 % of chest compressions exceeded a depth of 50 mm, with the mean (±standard deviation) compression rate of 127 ± 17 compressions per minute. Notably there were no misplacement of hands in the standard position, one hand misplacement in the over-the-head technique and three hand misplacements in the straddled technique. Chest releases proved to be insufficient in 43 % of cases across all positions, with pauses in chest compressions exceeding nine seconds observed in 36.9 % of instances, regardless of position or ventilation method.

Overall, the study underscores that rescuer position exerts limited influence on CPR quality. Across all positions, compressions were frequently too rapid, chest releases inadequate, and hands-off times often excessive. Even tidal volumes were consistently below recommended thresholds, suggesting areas for improvement in CPR practices irrespective of positioning.

### Confined space

**Fig. 11:**
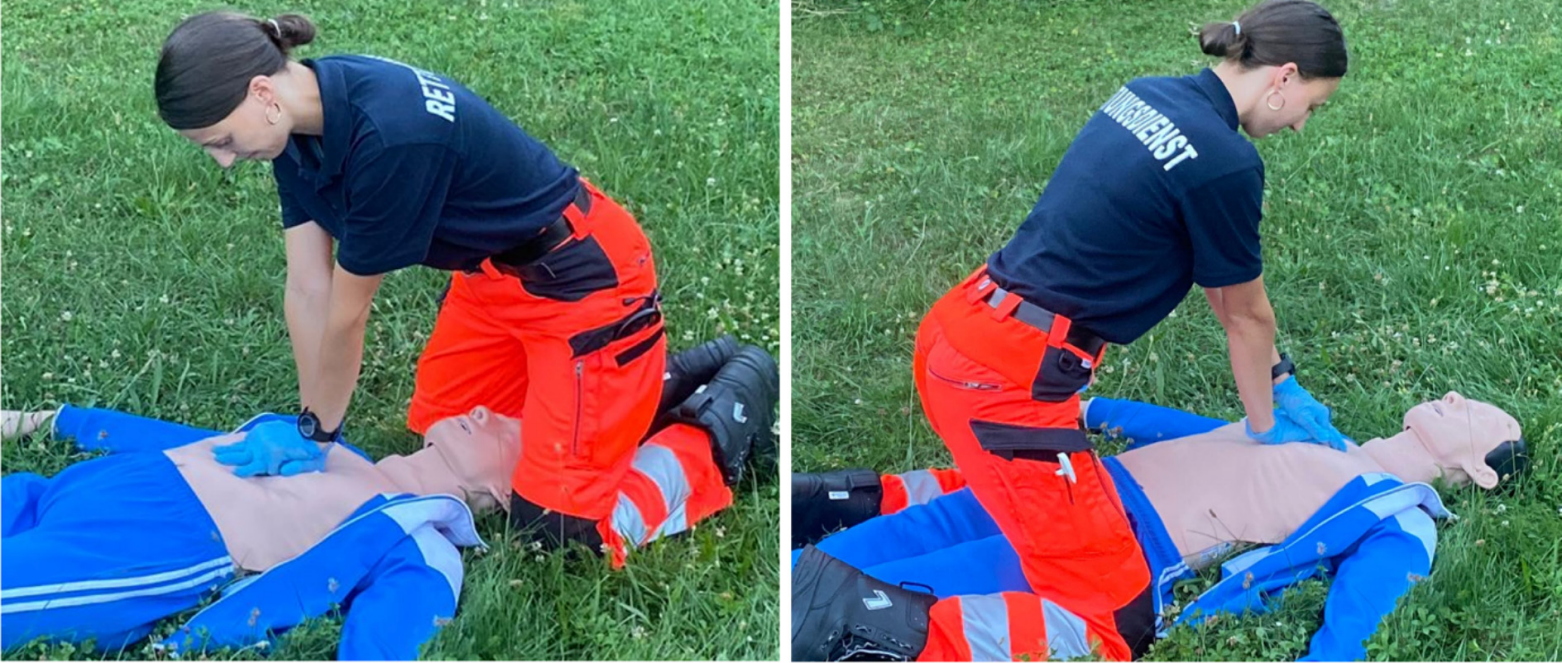
The OTH technique (left) and the straddle technique (right).

While other sections of this review focus on specific examples of extreme environments that are often associated with confined spaces, there are other ways to perform CPR in the prehospital setting under difficult spatial conditions. In this context, two CPR methods are at the centre of the investigations: The over the head technique (OTH) and the straddle technique (Fig. 11). We found eleven publications of CPR in restricted spatial conditions, all of which are manikin studies.

9 studies investigated the OTH technique. Bollig et al. and Chi et al. found no differences between the standard CPR and the OTH method in terms of kinematics, compression forces, compression depths and compression frequencies. Therefore, their studies attest the OTH technique to be comparable to the standard method.^22^, ^24^ Brucke et al. investigated the OTH method in a two-rescuer feasibility manikin simulation study. Their algorithm is designed to reduce the number of rescuers required while minimising this impact on CPR quality. According to Brucke et al., this is achieved in 2 phases: In the first phase, one rescuer performs OTH CPR and ventilation. The second rescuer is at the patient’s side, applies the defibrillator pads to the chest, activates the defibrillator and prepares the tracheal intubation and medication. In the second phase, the assistant standing to the side then takes over the compressions. Both rescuers can then take turns with chest compressions and ventilation in order to ensure uninterrupted care. Hereby, the authors proved the feasibility of their concept and call for further studies to evaluate the combination of standard CPR and OTH CPR.^23^

Ćwiertnia et al., Hüpfl et al., Maisch et al. and Perkins et al. investigated the OTH method in a single rescuer setting.^25^, ^27^, ^28^, ^30^ All concluded that OTH CPR offers a sufficient if not better alternative to the standard method. According to Ćwiertnia et al., OTH CPR showed shorter interruptions between compression cycles, a higher compression depth, a more correct compression rate as well as a more complete chest wall recirculation.^25^ On the other hand, Hüpfl et al. demonstrated shorter interruptions between chest compressions and significantly greater compression depth for the OTH method compared to the conventional method. Additionally, the compression rate was higher in the OTH group, and complete chest recovery after each compression occurred more frequently with the OTH method.

Maisch et al. compared three positions during CPR performed by a single rescuer using a bag-valve-mask device: OTH CPR, lateral CPR, and alternating CPR. Thus, OTH CPR was at least equivalent to lateral and superior to alternating CPR. Due to potential difficulties in bag-valve-mask ventilation in the lateral position, the authors recommend OTH CPR. Additionally, dual-operator CPR was significantly superior to all single-rescuer methods.^28^ In contrast, one-year earlier also Maisch et al. compared 2-rescuer OTH CPR versus lateral CPR,^29^ demonstrating shorter hands-off times over a monitored 2-minute period (25vs38s), significantly more chest compressions (167 vs 142 chest compressions/minute) and significantly more correctly performed chest compressions (72 vs 45 correct chest compressions) with the lateral CPR compared to OTH CPR. In a recent study, Wirmando et al.^32^ compared one-rescuer CPR in the traditional lateral position with CPR int the OTH position. The findings indicate that CPR in the OTH position results in fewer interruptions and better compression rates compared to the traditional lateral position, while the compression depth remains equal between the two methods.

Two studies were identified examining the straddle position. Handley et al. compared the quality of the straddle technique with the standard technique and the OTH-technique in a one- and two-person scenario.^26^ The results show that the straddle method for two-person CPR is comparable to the standard method and can be useful in confined spaces. However, in the one-person OTH method, hand placement was often inaccurate, which requires further studies to assess safety. In general, the study emphasises that with two trained rescuers, two-person CPR has advantages over one-person CPR, particularly in minimising pauses in chest compression. A study by Supatanakij et al. came to a similar conclusion, showing no significant differences between the straddle technique and conventional chest compressions in terms of compression rate, compression depth and rescuers’ fatigue.^31^

## Discussion

This scoping review identified 26 publications across six distinct extreme settings. The studies investigated alternative chest compression techniques using manikins to simulate cardiac arrest scenarios. Study designs varied, ranging from studies with one to 124 participants. Notably, there was considerable heterogeneity in how the population, intervention, comparators, and outcomes were defined and reported across these environments.

All studies focused on evaluating chest compression quality by assessing compression depth and compression rate. Alternative techniques were particularly prevalent in microgravity environments, where traditional methods are impractical. Although there have been no reported cardiac arrests in space to date, the risk of severe medical events occurring during long-duration spaceflights is a major concern. Recent guidelines have incorporated many alternative techniques into their recommendations,^5^ with the exception of the new Schmitz-Hinkelbein and the Cologne method, which may be considered for future updates.^14^ In order to provide high-quality CPR in space, a combination of different methods can be applied. The Schmitz-Hinkelbein-, ER- and Handstand method seem to have some advantages and can be applied as a first-approach method since chest compressions can be conducted immediately without any equipment until a crew-restraint-system is available.

In hypogravity, the reduced gravitational force increases the physical demands of traditional chest compressions, although they remain feasible.^20^, ^37^, ^38^ In these conditions, elbow flexion increases, and upper limb muscles actively engage to counterbalance the reduction in weight. This engagement is crucial as it prevents the rescuer from being inadvertently pushed away from the victim.^18^, ^39^ A recent systematic review has underscored the potential benefits of alternative rescuer’s positions during chest compressions in hypogravity conditions.^4^ This review identified two alternative techniques in hypogravity, both demonstrating superior CPR quality compared to traditional methods.^9^, ^11^ As the commercial space flight industry grows and crewed expeditions to the Moon and Mars become imminent, the likelihood of emergencies requiring CPR in space will rise. Consequently, there is an increasing need for the development of comprehensive basic and advanced life support protocols tailored to these extraterrestrial environments. To date, definite guidelines for CPR in hypogravity are missing.

While the lack of gravity is the major concern for adequate CPR quality in space, CPR in aeroplanes is characterized by two specific settings: A lack of space and the small potential of reliably recruiting medical personnel. Moreover, the opportunity to establish a complete intensive care environment can be delayed for several hours. Thus, CPR techniques in aeroplanes must be executable in confined space and by aeroplane staff with no or minor medical background. We found one study evaluating a new MRD. CPR was feasible for flight attendants using the new MRD, though ventilation quality dropped. The authors requested more tests on MRDs in aircrafts in order to be able to assess the safety of such systems in more detail.^21^

Similar problems exist for CPR in helicopters, where traditional CPR methods face challenges such as low headroom. In such settings, exploration also prompts into alternative approaches to optimize resuscitation efforts.^40^, ^41^ The Koch method was identified, showing superior CPR quality compared to traditional methods until mechanical systems become available.^33^

Challenges in out-of-hospital CPR scenarios due to limited space were addressed by the OTH and straddle techniques, enabling rescuers to initiate chest compressions in confined spaces. Eleven studies were found evaluating the efficiency of these techniques, demonstrating at least comparable CPR quality to traditional methods. These techniques are applicable in various constrained settings, including helicopters, boats, or aeroplanes, where space limitations are a concern.

In-water-resuscitation is challenging to perform due to the simultaneous stabilization of the victim’s body and concurrent chest compressions and/or ventilation. None of the manuscripts with focus on new techniques concerning thoracic compressions met the specifically predefined inclusion criteria. Although some studies have shown improved outcomes for patients when resuscitation was initiated in water, these mainly involved ventilation without chest compressions, as hypoxia is the primary cause of cardiac arrests in drowning cases.^8^ Winkel et al. showed that in-water resuscitation including advanced airway management, mechanical ventilation and automated chest compressions appear possible in a special self-inflating helicopter rescue platform.^42^ DuCanto et al. also showed feasibility of mechanic thoracic compressions using the air-driven device LUCAS for underwater compressions up to a depth of 10 meters.^43^

No studies were found that present new techniques for thoracic compressions on boats, where CPR can also be challenging due to varying boat sizes, space constraints, and wave conditions. Another challenging setting for delivering high quality CPR are avalanche scenarios. The poignant reality of nearly 150 fatalities annually in North America and Europe represents the victims killed by avalanches. This number may be significantly higher in developing countries.^44^ In comparison to 2006, where the number of victims killed by avalanches totalled around 140, this number has slightly increased. Of these, approximately 100 died in the European Alps, while five were reported in other European countries and 35 in North America.^45^

In situations where individuals are completely buried by avalanches, self-rescue is usually impossible. This greatly increases the urgency and complexity of rescue missions. Rescuers face a myriad of obstacles, from navigating rough and indistinct terrain to locating the injured individuals, who may be entirely submerged, possibly in a prone or overhead position, amidst harsh environmental conditions marked by cold temperatures.^46^ Extricating submerged individuals is often strenuous and technically complex. In these difficult circumstances, there arises a pressing need to adapt rescue techniques to suit the exigencies of the situation. This might include initiating resuscitation in the prone position to shorten the no-flow time during evacuation and transport in the supine position.

In instances where the rescued individual is successfully extricated, and a breathing cavity is established with a clear or potentially clear airway, prompt evacuation to lower altitudes with ongoing cardiopulmonary resuscitation (CPR) becomes imperative, particularly in cases of hypothermia, necessitating subsequent clinical intervention.^45^

Regarding avalanche scenarios, rescuer position appeared to have limited influence on CPR quality. Both OTH and straddle positions seem as effective as traditional CPR.^34^ However, for cases of prolonged CPR due to factors like hypothermia,^47^ CPR performed in the straddle position on a moving toboggan appeared to be less effective.^35^ The urgent need to transport the rescued patient for further clinical treatment necessitates deviation from the observed quality standards. Mechanical resuscitation aids might compensate for the deficits of manual resuscitation during transport, but studies are missing. During transport, Abrams et al. decided to move the patient in a “heads-up” position on the stretcher. Recent studies investigating heads-up CPR have shown better outcomes than CPR performed in the supine position due to the preservation of mean arterial pressure, decreased intracranial pressure and improved cerebral perfusion pressure.^48^ Though the current evidence for heads-up CPR is limited and does not support widespread adoption,^49^ it might become relevant in the future, especially in difficult CPR scenarios.

Even though the use of diving bells and hyperbaric chambers has a very limited scope, both settings also represent extreme environmental conditions. Thus, in a medical emergency, it can be necessary to adapt the applied resuscitation technique to the altered environment and space. No studies were included due to lack of presenting new CPR-techniques with evidence. Nevertheless, two techniques are to be mentioned in this context, although their presentation was devoid scientific evidence. Bhutani et al. present two techniques: the 'head-to-chest' technique involves pressing the forehead of the helper against the victim's sternum, thereby compressing the thorax. In the second method, known as the “knee-to-chest” technique, the helper utilizes their knee to compress the sternum of the victim.^50^ A recent comprehensive review recommends alternative chest compressions techniques in a hyperbaric chamber, like the straddle or OTH position, if spatial conditions make traditional CPR impossible.^51^ There are no studies investigating the efficiency of these techniques for these scenarios.

### Limitations

The limitations of this review include its’ focus on peer-reviewed publications while excluding grey literature, the absence of a formal assessment of the certainty of evidence and the lack of a published study protocol. Additionally, the screening was restricted to two databases. As no assessment of literature quality has been performed, it is not possible to draw definite practice-related recommendations from these results.

## Conclusions

To address the unique challenges in extreme environments, novel CPR techniques are emerging. The straddle and OTH method seem to be equal to the traditional technique and may be applied in all areas of confined spaces. In extreme environments like avalanches, hypogravity and drowning evidence remains limited. Continued research is crucial to develop and validate CPR protocols tailored to each extreme environment, ensuring optimal resuscitation outcomes in challenging circumstances.

## Ethics approval

Not applicable.

## Funding

Open Access funding enabled and organized by Projekt Deal.

## CRediT authorship contribution statement

**Remco Overbeek:** Writing – review & editing, Writing – original draft, Methodology, Investigation, Conceptualization. **Felix Liebold:** Writing – review & editing, Writing – original draft, Methodology, Investigation, Conceptualization. **Lydia Johnson Kolaparambil Varghese:** Writing – review & editing, Writing – original draft. **Niels-Benjamin Adams:** Writing – review & editing, Writing – original draft. **Jan Schmitz:** Writing – review & editing, Writing – original draft. **Michael Neumann:** Writing – review & editing, Writing – original draft. **Fabian Dusse:** Writing – review & editing. **Sandra E. Stoll:** . **Wolfgang A. Wetsch:** Writing – review & editing, Supervision. **Jochen Hinkelbein:** Writing – review & editing, Supervision, Conceptualization.

## Declaration of competing interest

The authors declare that they have no known competing financial interests or personal relationships that could have appeared to influence the work reported in this paper.
